# Metaplastic Changes in Chronic Cholecystitis: Implications for Early Diagnosis and Surgical Intervention to Prevent the Gallbladder Metaplasia-Dysplasia-Carcinoma Sequence

**DOI:** 10.4021/jocmr1689w

**Published:** 2013-12-13

**Authors:** Charalampos Seretis, Emmanouil Lagoudianakis, George Gemenetzis, Fotios Seretis, Apostolos Pappas, Stavros Gourgiotis

**Affiliations:** a Department of General Surgery, 401 General Army Hospital of Athens, Greece; b Medical School of Patras, Greece; c Department of Internal Medicine, Argos District Hospital, Greece

**Keywords:** Gallbladder, Cancer, Metaplasia, Cholecystectomy

## Abstract

**Background:**

Metaplastic features of the gallbladder epithelium are considered to be the precursors of gallbladder cancer. Considering the possible role of chronic inflammatory changes in the development of these lesions and the rationale for performing an early prophylactic cholecystectomy, we performed a retrospective study to assess the prevalence of gallbladder metaplasia in patients who underwent cholecystectomy due to underlying cholelithiasis.

**Methods:**

We reviewed the routine histopathology reports of 86 patients with chronic cholecystitis, who underwent elective cholecystectomy, to assess the prevalence of gallbladder metaplasia in the course of chronic cholecystitis. We further attempted to evaluate the existence of any correlations between the presence of the gallbladder metaplasia and the type of lithiasis, as well as the gallbladder wall thickness.

**Results:**

The overall prevalence of metaplastic features in the resected specimens was 25.6%. Dysplastic changes were more frequent in gallbladder specimens with concurrent metaplasia. Moreover, in presence of metaplastic changes, we observed an increase of the average gallbladder wall thickness. Finally, metaplastic and dysplastic changes were associated with the presence of micro-lithiasis rather than macro-lithiasis.

**Conclusions:**

Gallbladder metaplastic changes appear to be more frequent in cases of micro-lithiasis and seem to be associated with a chronic thickening of the gallbladder wall. Taking into account the usually sub-clinical course of this group of patients, when compared to patients with macro-lithiasis, further studies are needed to evaluate a possible role of prophylactic cholecystectomy in this population to prevent the long term evolution of these early changes to cancerous lesions.

## Introduction

Despite the tremendous progress in confronting gastrointestinal cancers, gallbladder cancer still remains a type of malignancy with a very poor prognosis [1]. Apart from its aggressive biologic behavior, the complete lack of screening methods and reliable biomarkers that would enable its early detection both contribute to a usually delayed diagnosis of gallbladder cancer, which on many occasions is an incidental finding after the performance of cholecystectomy. With the details of its natural course being still in the shades, gallbladder cancer is considered to initiate from long-standing metaplastic lesions, which sequentially become dysplastic before the final development of cancerous features and properties [2]. It has been proposed that the chronic inflammatory reaction of the gallbladder epithelium to the presence of gallstones contributes significantly to the progression, if not in the development as well, of these precursor lesions, in the framework of a globally accepted concept of the firm association between cancer growth and inflammation [3, 4]. Other factors probably triggering the early neoplastic changes of the gallbladder are the presence of anatomic variations, such as the pancreaticobiliary maljunction, which enables the free reflux of the pancreatic enzymes into the biliary system, and the colonization of the biliary tree by infectious agents, with the various Helicobacter species being a well-studied example [5, 6].

Therefore, it would be reasonable to assume that the recurrent episodes of cholecystitis could be landmarks in the development of gallbladder carcinogenesis. Considering that the performance of cholecystectomy is the only definitive solution on this occasion, it would be very tempting to pose the question whether an early cholecystectomy would be an effective way to prevent the occurrence of a malignancy that will become evident either incidentally or at an advanced stage, leaving no other option apart from palliative treatment.

In the common clinical practice, upon the clinical manifestation of cholecystitis and after the performance of the common radiological investigations, the patient is usually advised to either proceed with cholecystectomy or is guided to adopt healthy lifestyle modifications and re-present when symptoms re-occur to consider surgery. No matter how reasonable the above mentioned sound, with the option of surgery being offered in symptomatic cases, many questions arise regarding the possibility of evolving pre-cancerous changes in asymptomatic patients, who have for instance micro-lithiasis, which is less likely to cause significant obstructive phenomena, while it exhibits a prolonged inflammatory effect on the gallbladder epithelium. In the absence of biomarkers and sophisticated imaging techniques that would enable the detection of early pre-cancerous lesions in the gallbladder, it would be interesting to evaluate if the type of lithiasis (micro- or macro-lithiasis), as well as the thickness of the gallbladder wall, which is ultrasonographically measurable and is positively co-related with the degree of the chronic inflammatory changes [7, 8], could be associated with the presence of precursor lesions of the gallbladder cancer and could be used as possible “red flags” of these early changes.

## Methods

We performed a retrospective study, in which we included male patients with chronic cholecystitis who underwent elective cholecystectomy for cholelithiasis in our Institution. We excluded cases in which the histopathological reports were suggestive of acalculus cholecystitis, porcelain gallbladder, as well as cases where the resected gallbladder was opened intra-operatively; a total of 86 histopathology reports were included in the final study sample. The principal parameter evaluated was the presence or absence of metaplastic changes in the gallbladder epithelium and the latter was correlated with the patients’ type of lithiasis (micro-/macro-lithiasis) and thickness of the gallbladder wall. The histopathological examination of the resected specimens had been performed by two experienced Histopathology Consultants.

## Results

The histopathological examination revealed an overall prevalence of metaplastic features in the resected gallbladder specimens of 25.6% (22/86). In the presence of metaplastic features, there was a 40.9% (9/22) of accompanying dysplastic changes of pyloric (8/22) and intestinal type (1/22), while on one occasion the histopathological examination revealed the presence of gallbladder adenocarcinoma (1/22). Therefore, in total, the existence of metaplastic changes in the gallbladder epithelium was accompanied in 45.5% (10/22) of cases with dysplastic-cancerous lesions. Remarkably, in the absence of metaplastic features, no dysplastic or cancerous lesions were identified after the routine histopathological examination of the specimens.

When assessing the existence of potential differences regarding the thickness of the gallbladder wall when stratifying the patients according to the presence or absence of metaplastic changes, in cases with gallbladder metaplasia the average thickness of the gallbladder wall was 45 mm, while in the non-metaplasia group, the average thickness was 38 mm (45 ± 10 mm vs. 38 ± 11 mm).

Finally, in cases of gallbladder metaplasia, the underlying lithiasis type was micro-lithiasis in 81.8% (18/22) and macro-lithiasis in 18.2% (4/22) of the specimens; concerning the number of distinct gallstones in macro-lithiasis in this group of patients, in all cases this comprised of the presence of a sole gallstone. On the contrary, in the absence of gallbladder metaplasia, micro-lithiasis was reported in 59.4% (38/64) of cases, while in 29.7% (19/64) a sole gallstone was detected and in the remaining 10.9% (7/64) of cases multiple gallstones were present.

## Discussion

The absence of reliable biomarkers and screening examinations for gallbladder cancer, in combination with the biologically aggressive behavior of this type of malignancy result in poor prognosis for these patients. Considering the close interplay between chronic inflammation cancer development and its further loco-regional and metastatic progression, it would be reasonable to assume that chronic cholecystitis may be a major factor triggering early metaplastic changes in the gallbladder epithelium, which can gradually progress to dysplastic and finally cancerous lesions. This metaplasia-dysplasia-carcinoma sequence has been well-established as a concept in gallbladder cancer development, with the gross time framework for the actual acquisition of cancerous features being estimated at 10 years [3]. Therefore, an early surgical intervention, especially in young patients, could probably prevent this catastrophic scenario.

Undoubtedly, the current practice is to offer a surgical treatment with performance of cholecystectomy upon the occurrence of the relevant symptoms. Obviously, the cholecystectomy in the symptomatic group of patients will usually eliminate the long-term risk of gallbladder cancer before the occurrence of precursor lesions. However it is of paramount importance to consider that cholelithiasis is more likely to become symptomatic in cases of macro-lithiasis, where the obstructive symptoms are expected to be more profound, while micro-lithiasis can be subclinical for a more prolonged time period.

The overall prevalence of metaplastic changes in the gallbladder epithelium in our study sample is in alignment with the previously published data [9-11]. However, the present study is the first to demonstrate that early metaplastic changes in the gallbladder epithelium could be more frequent in patients with micro-lithiasis, posing therefore an important question about the role of preventive cholecystectomy in this group of patients, for instance after incidental detection of micro-lithiasis in abdominal imaging investigations, especially in young patients. Previous studies have reported a positive co-relation between the total amount, weight and size of the gallstones with the presence of concurrent metaplastic and dysplastic lesions of the gallbladder [12]; however, to the best of our knowledge, there are no other data examining this question in a “dichotomous” way.

Applying the above mentioned in a frequent clinical scenario, in which a patient is suspected to have cholelithiasis, but the imaging investigations demonstrate mild micro-lithiasis, the treating physician would probably discuss the need for an operation in the long-term and most likely the patient would be advised that future attacks could be prevented or postponed with appropriate lifestyle modifications. Our preliminary findings indicate that on this occasion, it is not unlikely that precursor lesions of gallbladder cancer may be already present, remarkably more frequent in cases of micro-lithiasis rather than macro-lithiasis. A reasonable assumption explaining the latter is that micro-lithiasis interacts with a greater surface of the gallbladder epithelium, causing in the long-term diffuse, low-grade pro-inflammatory changes, leading to sequential generalized de-arrangement of the local immune-surveillance mechanisms, favoring the development of metaplastic and dysplastic lesions. In indirect confirmation of our rationale was the increased gallbladder wall thickness in cases of present metaplastic features, indicating that the chronic inflammatory reaction to micro-lithiasis and the sequential fibrotic changes can provide a prosper soil for the development of gallbladder cancer.

Our study has significant limitations. First, the sample of our study comprised only of male patients, due to the nature of the hospital (military hospital), prohibiting the evaluation of sex-related differentiations in our concept. Moreover, our study is retrospective carrying the relevant risk of selection bias and the total sample of the patients included is rather small, not allowing safe statistical interpretation of our results.

Despite these limitations, to the best of our knowledge, our study was the only one to assess a possible association of the gallbladder wall thickness and the prevalence of metaplastic features in the gallbladder epithelium, fact that could be of great importance in terms of early identification of patients that are of higher risk of having a precursor lesion, as the gallbladder wall can be estimated by the routine ultrasonographic imaging. Finally, we demonstrated for the first time that micro-lithiasis is more likely to be associated with metaplastic changes in the gallbladder when compared to macro-lithiasis.

## Conclusions

Metaplastic changes in the setting of chronic cholecystitis are not infrequent, especially in patients with micro-lithiasis. Micro-lithiasis appears to be more frequently associated with concurrent metaplastic and dysplastic lesions when compared to macro-lithiasis, which could be theoretically attributed to a prolonged low-grade but more diffuse negative impact on the gallbladder epithelial cells. Macroscopically, these early changes seem to correlate with the presence of a thicker gallbladder wall, implicating a possible role for stratification of high-risk patients with the routine ultrasonographic evaluation, especially in the absence of clinical symptoms that would direct the clinicians towards the performance of cholecystectomy at an early stage.
